# Effect of autogenous osteochondral mosaicplasty on the balance control of patients with cartilage defects of the knee: a pilot study

**DOI:** 10.1186/s13018-023-03821-6

**Published:** 2023-05-06

**Authors:** Hai Tao, Yingchun Zhao, Fenghua Tao, Wei Xiang, Hui Cao, Zheng Zhang

**Affiliations:** Department of Orthopedics, Renmin Hospital of Wuhan University, 238, Jiefang Road, Wuchang District, Wuhan, 430060 Hubei China

**Keywords:** Balance control, Cartilage defect, Autogenous osteochondral mosaicplasty, Posturography

## Abstract

**Background:**

Autogenous osteochondral mosaicplasty (AOM) is a widely used optimal surgical technique for cartilage repair in young patients with focal articular cartilage defects. However, the alterations in balance control in these patients after AOM have not been sufficiently investigated. This study aimed to compare different balance control performances between the patients with knee cartilage defects and healthy controls before and after AOM, as well as evaluate the influence of AOM on balance control in these patients.

**Methods:**

Static posturographic tests were performed in twenty-four patients who were scheduled for AOM two weeks pre-, three months, and one year postoperatively, along with thirty matched controls, respectively. All participants underwent posturography under four standing conditions: eyes open and closed, without and with foam support to assess the balance control ability. Subsequently, patient-reported outcome measures (PROMs) were synchronously obtained and analyzed.

**Results:**

Compared to the control subjects, less efficient balance control was observed in study patients at three testing phases (*p* < 0.05), whereas no alterations in postural control were visible in these patients within a year following AOM (*p* > 0.05). Significant improvements were found in all PROMs such as the International Knee Documentation Committee, the Lysholm Knee Score, and the visual analogue scale in the study patients postoperatively (*p* < 0.01).

**Conclusion:**

The results indicated that patients with knee cartilage defects have a prominent balance control deficit compared to healthy individuals. Furthermore, AOM does not improve balance control in these patients for at least one year postoperatively, and more effective approaches for postural regulation are required for the management of cartilage defect patients.

## Introduction

Articular cartilage defect of the knee is a common reason for pain, limited mobility, and joint disability in orthopedics and sports medicine. The articular cartilage’s regeneration remains a significant challenge to date due to its avascular characteristics, making it difficult for nutrients and regenerative stimuli to penetrate the injured area [1]. With the growing awareness of the natural morphology and physiological properties of articular cartilage, it is now known that cartilage defects and osteoarthritis (OA) have evolved as a disorder of the entire osteochondral unit rather than a disease limited to surface cartilage [2]. As one of the most promising solutions, autogenous osteochondral mosaicplasty (AOM) is now being used as a local autograft to repair cartilage within current clinical therapeutic methods that transplanted healthy osteochondral plugs from the non-weight-bearing donor sites to the weight-bearing recipient zone [3, 4]. Thus, AOM aims to restore joint forms and facilitate the formation of healing surroundings, thereby reducing pain and improving knee function.

Balance control, an essential part of many ordinary activities, is ensured through the mechanism of postural maintenance and stabilization, requiring body orientation management in space by central nervous processing of visual, vestibular, and proprioceptive sensory inputs [5]. For maintaining a stable posture in daily life, the knee joint plays an important role as a neurosensorial structure as it contains muscles and ligaments that offer sensorimotor afference by involving several mechanoreceptors, that contribute to stabilizing the joint, and consequently the postural control [6]. Although inefficient balance control was observed in knee OA patients [7, 8], to the best of our knowledge, few studies focused on postural stability in patients with knee cartilage defects. The effect of autografts on balance control and activity-related functions following osteochondral transplantation in these patients remains ambiguous. Hence, comparing the differences in balance control abilities between patients with cartilage defects and healthy individuals, as well as exploring the potential postural stability changes in patients postoperatively, seems critical in the management of cartilage injuries, not only for their daily activities but also for rehabilitation progress following surgical interventions.

This study aimed to compare the different balance control performances between knee cartilage defect patients and control subjects before and after AOM. We also suggested that the restored quality of equilibrium might be comparable to that of healthy individuals. The secondary aim was to compare the alterations of balance control in normal and disturbed conditions in patients before and after the AOM. Thus, we advocated that these patients may have improved postural stability postoperatively due to structural recovery of chondral forms.

## Materials and methods

### Participants

Patients with knee joint pain and cartilage defects proved by magnetic resonance imaging (MRI) were recruited from the outpatient clinic of Renmin Hospital of Wuhan University and scheduled for AOM. Patients were only included if a 1.0–4.0 cm^2^ chondral defect and a ≥ grade 3 of Outerbridge classification score were confirmed in a unilateral knee by a preliminary arthroscopy performed by the same surgeon [9, 10]. Exclusion criteria were: participants with > 8 cm^2^ chondral defect, cartilage defects in both of the knees, joint infection, tumor, rheumatoid arthritis, arthroplasty in lower extremities, MRI-proven ligament injuries, uncontrollable joint pain, other musculoskeletal disorders, dysopia, neurologic impairment, and severe depressive syndromes. Moreover, patients > 50 years, with a 4–8 cm^2^ chondral defect or a ≥ grade 3 of Kellgren–Lawrence OA classification score, were not recommended for AOM [11]. Finally, 24 patients (15 and 9 individuals having manifestations in the left and right knees) meeting the inclusion criteria were scheduled for AOM. Furthermore, 30 control subjects with no lower limb pathology or a knee trauma history were recruited from hospital staff, students and local communities using advertisements.

The protocol and design of this observational study were reviewed and approved by the medical ethical committee of Renmin Hospital of Wuhan University. Written informed consent was obtained from each subject before participation. The characteristics of participants are summarized in Table 1. There were no significant differences in clinical variables between the knee cartilage defect patients and control subjects.

**Table 1:** Characteristics of participants

	Patients (*n* = 24)	Control subjects (*n* = 30)	*χ*^2^ test *p*-value
Sex (male/female), *n* (%)	16/8 (67/33)	19/11 (63/37)	0.362
Age (years), mean (SD)	34.7 (3.8)	36.4 (5.1)	0.506
Height (cm), mean (SD)	167.6 (6.3)	170.2 (4.6)	0.473
Weight (kg), mean (SD)	74.8 (7.9)	76.5 (6.2)	0.615
BMI (kg/m^2^), mean (SD)	27.1 (3.4)	26.7 (3.8)	0.527
Recipient site, *n*			
Medial femoral condyle	13	–	
Lateral femoral condyle	11	–	
Lesion size (cm^2^), mean (SD)	2.8 (0.9)	–	

### Experimental protocol

After the preliminary arthroscopic examination, the size and localization of the defect, number as well as size of autografts were recorded and assessed for AOM. Patients underwent the posturographic tests two weeks pre-, three months and one year postoperatively, respectively. Control subjects were measured three times at the same time and place as the study patients. Patient-reported outcome measures (PROMs) were obtained after all patients underwent posturography at each testing phase and included the International Knee Documentation Committee (IKDC) [12, 13], the Lysholm Knee Score [14], and the visual analogue scale (VAS) [15].

Various balance control performances between the study patients and control subjects, as well as the balance control variations in patients before and after AOM, were assessed. Meanwhile, the changes in PROMs in patients, along with the correlation between postural sway and PROMs, were also evaluated.

### Autogenous osteochondral mosaicplasty

All the AOMs were performed by the same surgeons with the standard open technique [16]. Grafts were harvested from the periphery of the non-weight-bearing sites including the lateral trochlea near the sulcus terminalis, intercondylar notch, and medial trochlea. A circular punch created sockets in the defect for the implantation of grafts at the recipient site. Grafts were spaced approximately 3 mm apart to avoid the confluence of tunnels.

Patients kept the knee in continuous extension with a brace for four weeks postoperatively and then began progressively non-weight-bearing functional exercises within the ensuing four weeks, like straight leg raising, isometric quadriceps, and continuous passive motion to avoid joint stiffness and muscular atrophy. Furthermore, crutches or walkers were subsequently used to facilitate weight-bearing from partial to full eight weeks postoperatively till the patient was walking independently without assistance. All the patients displayed cartilage healing, as seen by MRI and painless walking during the postoperative outpatient follow-ups. The incorporation of the bone plug into the native bone and the presence of a flush articular surface between the repaired and native cartilage were required in the postoperative MRI to judge successful cartilage healing. Finally, twenty-one and three patients reported complete osteochondral union at 3 and 4 months postoperatively, respectively.

### Posturography

All participants underwent balance control tests in a quiet and bright room in the hospital’s inpatient department for posturographic measurements and were measured by the same operator on a vertical force platform (Win-Posturo, Medicapteurs, Balma, France). The platform’s bottom was equipped with three strain-gauge force transducers, providing a measurement of the body sway in terms of displacement of the center of foot pressure (CoP) in a two-dimensional horizontal plane (recording time: 25.6 s, acquisition frequency: 40 Hz). The signals from transducers were amplified, converted from analog into digital form, and then recorded on a computer. The low postural sway area (in mm^2^) covered by the CoP trajectory represented good balance control precision [17]. Participants were asked to stand bare feet and upright on the platform with their feet abducted at 30°, heels apart by 3 cm, and arms along the sides, maintaining the body as stable as possible, and looking straight ahead at the center of a computer screen located at eye level 3 m away [18, 19]. Four conditions (C1–C4) involving two visual (eyes open and closed) and two platform (firm and foam support) conditions were provided during the test to imitate different sensory afferent environments for assessing their ability to effectively use sensory inputs and to suppress altered sensory information (Table 2). The basic measurements regarding the participant’s stability were taken on firm support with eyes open (C1) or closed (C2). Furthermore, a 10-cm-thick foam (Jinniu, JSC, Linyi, China) was subsequently placed on the platform to modify the somatosensory cues. It is suggested that enhanced measurements of balance control were implemented with eyes open (C3) or closed (C4) on foam support [20]. For each posturographic condition, three trials were conducted and a mean value of postural sway denoted the final result. For an accurate evaluation of the subjects’ ability to adapt and regulate balance control as per internal and external constraints, a mean equilibrium score (MES) was introduced by adding the individual condition scores and then dividing that sum by four [18].

**Table 2:** Determination of four testing conditions in balance control test

Conditions	Situation	Sensory consequences
Condition 1 (C1)	Eyes open, firm support	–
Condition 2 (C2)	Eyes closed, firm support	No vision
Condition 3 (C3)	Eyes open, foam support	Modified proprioception
Condition 4 (C4)	Eyes closed, foam support	No vision, modified proprioception

### Statistical analysis

All statistical analyses were performed using the SPSS software version 22.0 (IBM, Armonk, USA). The Kolmogorov–Smirnov test was used to check a normal distribution of quantitative variables. The qualitative data were displayed as a number (*n*) and percentage (%) and compared by *χ*^2^ tests. Postural sway in all conditions between three testing phases and PROM scores was compared by ANOVA test (normally distributed data) and followed by Bonferroni correction for post hoc comparisons. Subsequent divergences of balance control between study patients and control subjects were assessed by independent-samples *t*-test for normally distributed data. Pearson’s product–moment correlation coefficient was used for correlating MES at each testing phase along with the PROMs. All statistically significant differences were accepted for a probability level of *p* < 0.05.

## Results

### Differences in balance control between the study patients and control subjects

**Fig. 1:**
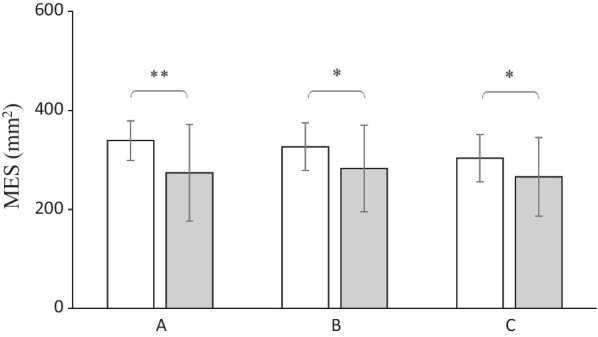
Mean values, associated with standard deviations, of the sway area (in mm^2^) for the mean equilibrium score (MES) observed in patients with cartilage defects (white bars) and control subjects (gray bars) 2 weeks before AOM (panel A), 3 months after AOM (panel B), and 12 months after AOM (panel C); *p < 0.05, **p < 0.01

Reflecting postural stability, the MES displayed significant heterogeneities between the study patients and healthy individuals preoperatively (*p* = 0.002), three months (*p* = 0.023), as well as one year postoperatively (*p* = 0.035) (Fig. 1). Although the study patients possessed a higher postural sway value (mean = 339.1 ± 40.0 mm^2^) than the control subjects before AOM (mean = 273.9 ± 97.8 mm^2^), with the autograft healing, the study patients still demonstrated more postural sway at three months (mean = 326.7 ± 48.0 mm^2^) compared to the control subjects (mean = 282.8 ± 87.5 mm^2^). One year postoperatively, these patients’ postural sway value (mean = 303.6 ± 47.6 mm^2^) remained higher when compared with the control subjects (mean = 265.7 ± 79.6 mm^2^). However, no differences were observed between the three measurements of the control subjects.

### Variation in balance control in patients with cartilage defects

**Fig. 2:**
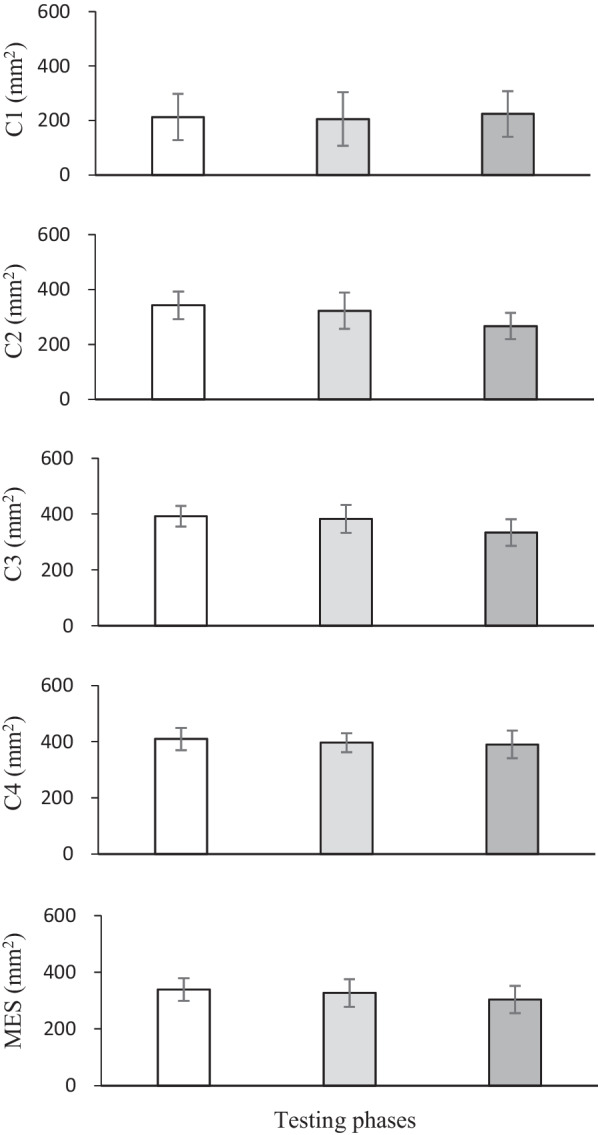
Mean values, associated with standard deviations, of the sway area (in mm^2^) for the four conditions (C1–C4) and the mean equilibrium score (MES) observed in patients with cartilage defects 2 weeks before AOM (white bars), 3 months after AOM (light gray bars), and 12 months after AOM (dark gray bars)

The variations in balance control in patients with cartilage defects in different conditions before and after AOM are presented in Fig. 2. No significant difference in postural stability was observed in C1 (*p* = 0.966). The patients displayed comparable postural sway two weeks preoperatively (mean = 212.0 ± 81.3 mm^2^) compared to the measurements at three months (mean = 205.1 ± 97.4 mm^2^) and one year postoperatively (mean = 223.5 ± 80.3 mm^2^). In the absence of vision, noticeable divergence was observed in C2 (*p* = 0.033). Patients displayed a lower sway area one year after AOM (mean = 266.4 ± 46.1 mm^2^) compared to that tested preoperatively (mean = 342.0 ± 48.4 mm^2^) (*p* = 0.032). However, this difference was not accepted by the Bonferroni correction for post hoc analysis (*p* > 0.05/3). The postural stability three months postoperatively (mean = 322.8 ± 63.2 mm^2^) did not change when compared to preoperative levels. As the somatosensory cues were modified, patients showed heterogeneity of balance control with vision availability in C3 (*p* = 0.039). A lower postural sway was found one year postoperatively (mean = 334.2 ± 45.9 mm^2^) in contrast to their preoperative levels (mean = 392.5 ± 35.7 mm^2^) (*p* = 0.045); the difference was not statistically significant, as proved by Bonferroni correction. Furthermore, the postural sway three months postoperatively (mean = 382.5 ± 48.2 mm^2^) remained similar to the preoperative values. In the vision-absent and proprioception-modified conditions of C4, no obvious alterations were found in these subjects before and after AOM (*p* = 0.292). Patients had a comparable postural sway at two weeks pre-(mean = 409.7 ± 39.1 mm^2^), three months (mean = 396.4 ± 32.3 mm^2^), and one year postoperatively (mean = 390.2 ± 48.0 mm^2^). Since the MES of postural sway, which reflects an overall balance control, did not show any significant difference (*p* = 0.261), the patients did not display a noticeably improved balance control performance three months (mean = 326.7 ± 48.0 mm^2^) and one year postoperatively (mean = 303.6 ± 47.6 mm^2^) when compared to their preoperative values (mean = 339.1 ± 40.0 mm^2^).

### Improvements in patient-reported outcome measures

**Fig. 3:**
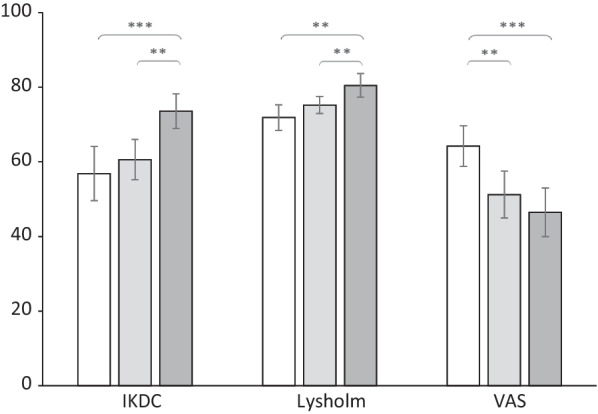
Mean values, associated with standard deviations, of patient-reported outcome measures observed in patients with cartilage defects 2 weeks before AOM (white bars), 3 months after AOM (light gray bars), and 12 months after AOM (dark gray bars); IKDC: The International Knee Documentation Committee scoring; Lysholm: The Lysholm Knee Score; VAS: the visual analogue scale; **p < 0.01, ***p < 0.001

The results of self-reported clinical outcomes in patients are shown in Fig. 3. Patients displayed higher IKDC scores one year postoperatively (mean = 73.6 ± 4.7) when compared to that measured two weeks pre-(mean = 56.9 ± 7.2) and three months postoperatively (mean = 60.6 ± 5.4) (*p* < 0.01). Similarly, the Lysholm scores displayed improvements likewise one year postoperatively (mean = 80.5 ± 3.2) in contrast to the preoperative (mean = 71.9 ± 3.4) and three-month postoperative values (mean = 75.3 ± 2.3) (*p* < 0.01). Knee joint pain reflected by VAS in patients exhibited relief three months (mean = 51.3 ± 6.3) and one year postoperatively (mean = 46.5 ± 6.5) when compared to their preoperative level (mean = 64.3 ± 5.4) (*p* < 0.01). Table 3 displays the outcomes of Pearson’s product–moment analysis comparing the posturographic evaluations and the PROMs. No significant correlations were found except for the postural sway and the VAS one year postoperatively (*r* = − 0.464, *p* < 0.05).

**Table 3:** Outcomes of Pearson’s analysis comparing the MES at each testing phase and the PROMs

	IKDC		Lysholm		VAS	
	*r*	*p*	*r*	*p*	*r*	*p*
Pre-AOM	0.759	0.056	0.156	0.466	− 0.296	0.160
Post-AOM_1_	0.022	0.920	0.288	0.172	− 0.076	0.723
Post-AOM_2_	0.402	0.051	0.192	0.368	− 0.464	0.022*

MES: mean equilibrium score; Pre-AOM: 2 weeks before AOM; Post-AOM_1_: 3 months after AOM; Post-AOM_2_: 12 months after AOM; *p ≤ 0.05; IKDC: The International Knee Documentation Committee scoring; Lysholm: The Lysholm Knee Score; VAS: the visual analogue scale

## Discussion

The results of posturography were not concordant with our hypotheses. Although less efficient balance control was observed in patients before AOM in comparison with the control subjects, this deficit was still present even after the structural restoration of articular cartilage. Moreover, patients with cartilage defects had no significant variation in balance control within a year following AOM; however, chondral autograft did not influence the postural stability of these study patients.

Injuries in different parts of the knee may give rise to different postural stability outcomes. Al-Dadah et al. [21] reported that the patients with isolated meniscal tears had a significant proprioceptive deficit after arthroscopic meniscectomy, which led to postural instability when compared to control subjects. Lion et al. compared the double-leg postural control under visual and surface perturbations of patients with anterior cruciate ligament (ACL) reconstruction with healthy controls and showed similar performance in postural control during double-leg stance in rehabilitated patients 6–14 months after ACL reconstruction [22]. Our study patients, even after undergoing AOM, had persistent balance control deficits when compared with control subjects within a year. We attempted to explain the various postural stability outcomes in patients with different knee injuries by analyzing the mechanical and biological properties of the joints. The avascular nature results in an inability of the cartilage to heal and predisposes the individual to knee instability [23]. Since several mechanoreceptors involving Pacinian corpuscles, Ruffini endings and Golgi organs are present within articular cartilage [24], numerous studies have reported that the number of mechanoreceptors decreases with age and is positively correlated with the level of proprioception in the knee joint [25, 26]. However, no relevant studies have quantified the mechanoreceptors in the meniscus, cruciate ligament, and cartilage to date. The ACL reconstruction may have retained relatively more mechanoreceptors than cartilage autografts and meniscectomy. Furthermore, patients with ACL reconstruction were younger (mean age = 24.9) as compared to the subjects with meniscectomy (mean age = 34) and our study patients (mean age = 34.7). Additionally, cartilage defects may cause joint pain, which can further exert a negative influence on knee proprioception and postural sway in knee OA patients [27–29]. Thus, ineffective cartilage recovery, damaged mechanoreceptors, joint pain, and age factor may incur irregular and uncontrolled sensory afference in the affected lower limbs, leading the patients to unstable posture while standing.

The articular cartilage interface and its supporting bone are tightly coupled and should be viewed as a connected osteochondral unit [30]. Although the biomechanical perturbations caused by osteochondral changes substantially alter the pattern and magnitude of contact forces and joint cartilage strains [31], AOM allows a replacement of the entire osteochondral structure, thus avoiding the potential side effects of altered subchondral bone on cell-based therapy procedures. Morphological restoration of cartilage has been addressed by an osteochondral autograft. However, a structural recovery does not imply complete functional restoration due to proprioception weakness, absence of vascularization, and neural ingrowth into the repaired cartilage, thereby resulting in joint instability and postural control deficit in the affected patients. Similarly, morphological restoration does not completely clear the proprioceptive disturbance emanating from the mechanoreceptors within the injured femoral condyle. Thus, it would be impossible to ensure a complete restoration of normal electrical impulses generated by implanted healthy mechanoreceptors in autograft plugs within a short time. This results in inefficient transmission of somatosensory information and poor utilization of anticipatory and compensatory strategies to modulate body equilibrium.

Conventionally, balance control during a quiet stance requires a particular sensorimotor strategy to regulate posture, which depends on the choice of visual or proprioceptive cues to perceive divergences between the planned and adopted postures [32]. For maintaining an upright stance in various environmental conditions, sensorimotor cues acquired from the surroundings should be selected by the central nervous system (CNS) so that prompt and accurate motor commands can be generated and conveyed to the muscles [33]. In our study, patients displayed an undifferentiated balance control performance pre- and postoperatively in the basic situation, in which available visual and proprioceptive afferent signals were captured by CNS, thus generating correct neuromuscular response and a stable posture. In the absence of vision, somatosensory inputs play a predominant role in spatial reorientation to maintain a high quality of equilibrium during static standing [34]. However, our study patients did not exhibit a more reasonable use of somatosensory information for maintaining balance and a more sensible shift of sensorimotor dominance from vision to proprioception postoperatively in the absence of visual reference.

Previous research revealed that the subjects rely more on proprioceptive information than visual and vestibular inputs to modulate body sway during static standing [35]. Due to the somatosensory afference disturbances, superficial plantar mechanoreceptors provide the CNS with inappropriate information relative to the body’s position and the vertical reference, which relies on gravitational forces, the reaction forces from the supporting surfaces [36]. Our foam support results did not show any noticeable improvements in patients’ balance control after AOM, thus indicating an unobvious recovery of the somatosensory system and inefficient regeneration of compensatory balance strategies. Overall, no differences were observed in patients with knee cartilage defects before and after AOM in any of the postural situations, irrespective of the testing conditions. The MES of patients revealed an undifferentiated postural sway within one year postoperatively, reflecting the indistinctive osteochondral autograft’s influence on balance control in individuals with cartilage defects.

In our study, PROMs displayed improvements after AOM, which were inconsistent with their subsistent balance control performances. Although pain, joint stiffness, and articular dexterity have been reported as significant predictors of postural sway in patients with knee disorders [18, 27], it is known that weight-bearing restriction and range-of-motion limitations are two of the most important aspects of creating an environment that facilitates cartilage healing procedures [37]. Controlled early partial to full weight-bearing offers favorable stimuli to the defect area and repaired site for autograft healing. Additionally, repeated stress stimuli provide biomechanical signals to promote matrix production that may facilitate tissue repair. Furthermore, postoperative early mobilization changes the pressure of the joint cavity and improves the exchange of nutrients between the synovial fluid and extracellular cartilage matrix. In this respect, our IKDC and Lysholm Knee Score outcomes demonstrated improved loading and mobility of the affected joint following AOM, thereby suggesting a functional rehabilitation of the knee joint that may provide potential benefits to the recovery of sensorimotor efficiency and the generation of compensatory balance strategy for postural regulation. Even if surgical inventions show no influence on patients’ postural stability within a year, improved knee mobility and clinical symptoms along with alleviated joint pain, despite limited relevance to the postural sway following AOM, provide confidence to the patients for addressing better balance control performance in later years.

There were also several limitations to this study. Although we excluded patients with bilateral cartilage defects and selected unilateral injury patients to avoid bias, the bilateral autograft may influence the balance control postoperatively in these patients. Furthermore, we did not extend the tests beyond one year postoperatively, so we were unable to assess future balance control variations in these patients and a potential alteration of postural stability in comparison with healthy individuals. Therefore, future studies should evaluate long-term balance control changes in patients with cartilage defects so that more inspiring outcomes may be discovered during postoperative convalescence.

## Conclusion

To conclude, this study substantiated the findings that patients with knee cartilage defects have a prominent balance control deficit as compared to normal individuals. Even if they undergo autografts, persistent reduced balance control ability suggests the AOM does not improve postural stability in these patients for at least one year postoperatively. Moreover, structural recovery of chondral forms cannot ensure adequate sensorimotor efficiency and the generation of anticipatory strategies while striving for balance control. However, improved self-reported clinical outcomes provide a potential for the restoration of balance control for longer periods postoperatively. Our study findings reveal that the knee joint needs further recovery of its corrective compensatory role in postural regulation following AOM and should be considered while managing patients with cartilage defects.

## Data Availability

All data generated and analyzed during this study are available from the corresponding author on reasonable request.

## Abbreviations

AOM: Autogenous osteochondral mosaicplasty
PROMs: Patient-reported outcome measures
OA: Osteoarthritis
MRI: Magnetic resonance imaging
IKDC: International Knee Documentation Committee
VAS: Visual analogue scale
CoP: Center of foot pressure
MES: Mean equilibrium score
ACL: Anterior cruciate ligament
CNS: Central nervous system
